# Modeling individual preferences reveals that face beauty is not universally perceived across cultures

**DOI:** 10.1016/j.cub.2021.03.013

**Published:** 2021-05-24

**Authors:** Jiayu Zhan, Meng Liu, Oliver G.B. Garrod, Christoph Daube, Robin A.A. Ince, Rachael E. Jack, Philippe G. Schyns

**Affiliations:** 1 Institute of Neuroscience and Psychology, University of Glasgow, Glasgow, Scotland G12 8QB, UK; 2 School of Psychology, University of Glasgow, Glasgow, Scotland G12 8QB, UK

**Keywords:** facial attractiveness, 3D face, reverse correlation, cultural diversity, individual preference, intelligent virtual agents

## Abstract

Facial attractiveness confers considerable advantages in social interactions,^1^^,^^2^ with preferences likely reflecting psychobiological mechanisms shaped by natural selection. Theories of universal beauty propose that attractive faces comprise features that are closer to the population average^3^ while optimizing sexual dimorphism.^4^ However, emerging evidence questions this model as an accurate representation of facial attractiveness,^5^, ^6^, ^7^ including representing the diversity of beauty preferences within and across cultures.^8^, ^9^, ^10^, ^11^, ^12^ Here, we demonstrate that Western Europeans (WEs) and East Asians (EAs) evaluate facial beauty using culture-specific features, contradicting theories of universality. With a data-driven method, we modeled, at both the individual and group levels, the attractive face features of young females (25 years old) in two matched groups each of 40 young male WE and EA participants. Specifically, we generated a broad range of same- and other-ethnicity female faces with naturally varying shapes and complexions. Participants rated each on attractiveness. We then reverse correlated the face features that drive perception of attractiveness in each participant. From these individual face models, we reconstructed a facial attractiveness representation space that explains preference variations. We show that facial attractiveness is distinct both from averageness and from sexual dimorphism in both cultures. Finally, we disentangled attractive face features into those shared across cultures, culture specific, and specific to individual participants, thereby revealing their diversity. Our results have direct theoretical and methodological impact for representing diversity in social perception and for the design of culturally and ethnically sensitive socially interactive digital agents.

## Highlights

• We modeled individual preferences for attractive faces in two cultures
• Attractive face features differ from the face average and sexual dimorphism
• Instead, culture and individual preferences shape attractive face features
• Attractive face features from a culture are used to judge other-ethnicity faces

## Teaser

Zhan et al. refute theories of universal beauty, showing that Western and Eastern cultures and individual preferences shape attractive face features. Individual preference models show that attractive features differ from the average and sexual dimorphism to form a space that cultural members use to perceive face attractiveness in other ethnicities.

## Results and discussion

To test universality, we started our analysis by modeling each participant’s preference—i.e., face features (i.e., 3D shape and L^∗^a^∗^b^∗^ complexion) that modulate perceptions of attractiveness. Using these 3D face models, we asked two key questions: is facial attractiveness a universal face average and is it an exaggeration of sexual dimorphism? Having shown that it is neither, we reconstructed a more-accurate representation of the feature space of facial attractiveness. Within it, we show that attractiveness preferences vary within and across cultures and that cultural preferences transfer to faces of other ethnicities.

### Modeling individual facial attractiveness preferences in two cultures

Specifically, we modeled individual’s preferences with young males (median age, 23 years old) known to rely on physical appearance when judging attractiveness^13^, ^14^, ^15^, ^16^ from two distinct cultures—i.e., 40 white Western Europeans (WEs) and 40 Chinese East Asians (EAs) (see Participants in the STAR Methods). We controlled face ethnicity as a between-participant factor, with half of the participants in each culture (i.e., 20 out of the 40 participants) rating faces of their own ethnicity and half the other ethnicity.

Our model construction extended beyond the common practice of computing group averages in experimental designs that focus on testing a specific hypothesis of a given theory. Instead, using a data-driven design, we modeled the subjective facial attractiveness preferences of each individual cultural participant. We also used naturally varying random 3D face stimuli synthesized by a generative model (henceforth, GMF; i.e., see 3D face stimuli in the STAR Methods). Specifically, the GMF^17^ models the 3D shape and complexion of each face stimulus as the sum of a categorical average component (i.e., with set factors of age: 25 years old, sex: female, ethnicity: WE or EA) plus a residual random component of parameters that control face identity. The GMF therefore accurately models and generates the natural variations of shape and complexion in the target populations (as demonstrated in Figure S1C).

In the experiment, each participant saw on each of 1,950 trials a randomly generated 3D face displayed in one of three viewpoints (−30°, 0°, and +30° of depth rotation). Participants rated its attractiveness on a 9-point scale ranging from 1, not attractive at all, to 9, very attractive (see Procedure in the STAR Methods). To model facial attractiveness, we linearly regressed the variations of each GMF identity parameter of shape and complexion across trials with the corresponding variations of the participant’s attractiveness ratings (ensuring that assumptions of linearity held; see Linear regression model in the STAR Methods). This produced a total of 80 individual 3D face models (i.e., 20 models of each observer-face ethnicity combinations) that we validated (see Model validation in the STAR Methods).

### Feature selection: Is attractiveness a universal face average?

To address this question, we computed in each culture the departure of the modeled face features of attractiveness from the average 25-year-old WE or EA female face. For fair comparison, we performed these analyses using group models, by averaging the individual models of the same-ethnicity conditions—i.e., 20 WE or EA participants rating WE or EA faces, henceforth “Western-same” or “Eastern-same.”

#### 3D face shape

**Figure 1:**
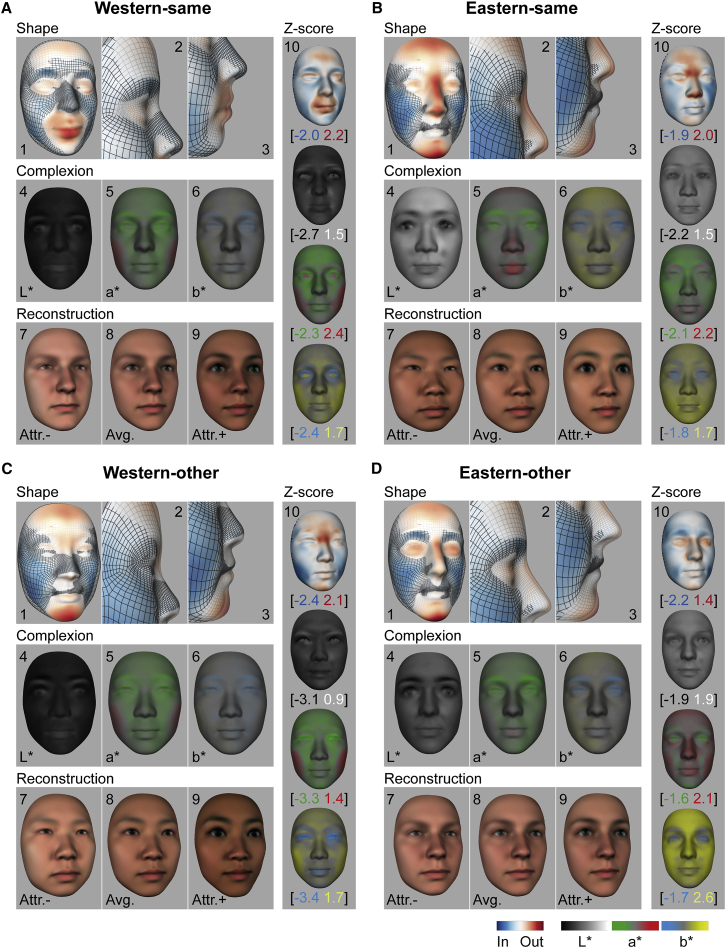
Female face features that modulate male cultural perceptions of attractiveness (A) Western-same. (1) Attractive shape features averaged across participants are shown. (2 and 3) Profile enlargements are shown. Attractive 3D shape features deviated outward (in red) and inward (in blue) from the 25-year-old WE female average shape (gray mesh). (4–6) Complexion is shown. Attractive L^∗^a^∗^b^∗^ deviations away from the 25-year-old WE female average complexion, averaged across participants, are shown. Colored scales on the face indicate the effect size of shape and complexion deviations (i.e., β slope of the linear regression models), normalized to the maximum of each shape display or across L^∗^a^∗^b^∗^ displays. (7 and 9) Unattractive and attractive 3D face reconstructions are shown as shape and complexion deviations from the 25-year-old WE female average (8). (10) *Z* scored shape and complexion attractive deviations with minima and maxima *Z* scores between brackets. Attr., attractive; Avg., average. See also Figure S2, Video S1, and Tables S1 and S2. (B) Eastern-same, same format as (A). (C) Western-other, same format as (A). (D) Eastern-other, same format as (A).

Figures 1A and 1B (panels 1–3) show how the Western-same and Eastern-same group models differed from their same-ethnicity averages. In each culture, attractive faces were systematically smaller than their respective categorical average (cf. blue codes inward deviations of the cheeks and jaw relative to the gray mesh average). They also had more prominent foreheads and rounder, protruding eyes (cf. red codes outward deviations). However, there were also marked cultural differences of an extruding (poutier) mouth shape in WEs and a higher nose bridge and pointier chin in EAs (see Figures 1A and 1B, panels 2 and 3; Figure S2 reports individual models).

#### Complexion

Figures 1A and 1B (panels 4–6) show how the Western-same and Eastern-same group models differed from their same-ethnicity averages. Both cultures preferred darker than average eyelids and eyelashes (i.e., at higher contrast). WEs preferred a darker (Figure 1A, panel 4) and redder (Figure 1A, panel 5) complexion than average, whereas EAs preferred lighter complexions (Figure 1B, panels 4 and 6) and redder lips (Figure 1B, panel 5; Figure S2 reports individual models).

To visualize the attractive and unattractive (i.e., opposite direction) faces, we added the same-ethnicity attractive/unattractive deviations to the average of each ethnic female face (see Figures 1A and 1B, panels 7–9; Video S1; and Reconstructing attractive and unattractive faces in the STAR Methods).

Video S1. Feature transitions along cultural facial attractiveness, related to Figure 1 The larger 3D face shows the reconstruction at different level of face attractiveness, from the female average to attractive female and to unattractive female. The smaller adjacent faces colored in scale the shape and L^∗^a^∗^b deviations of the face reconstruction from the female average.

### Feature selection: Is attractiveness distant from the face average?

To address this question, we *Z* scored the distribution of all randomly generated face variations that participants rated (see Face average and feature distribution in the STAR Methods). Figure 1, panel 10, shows the results. In both cultures, attractive face shape and complexion features sit at the outskirts of the distribution (i.e., >1.5 SD away from the sampling range of deviations), akin to a “hidden preference” in face processing mechanisms—i.e., a peak drift.^18^^,^^19^ Distant features comprise a pouty mouth in WEs and high nose bridge in EAs, both of which are popular in plastic surgery in each culture.^20^^,^^21^ For complexion, darker eye lids in both cultures reflect popular makeup choices; darker skin in WEs^22^ and lighter skin in EAs^22^^,^^23^ are also consistent with cultural cosmetic choices. In fact, these modeled features match those of 36 independent psychological, cosmetic, and plastic surgery reports (see Tables S1 and S2 for listings and our corresponding group and individual models in Figures 1 and S2, respectively).

### Feature selection: Does attractiveness exaggerate sexual dimorphism?

**Figure 2:**
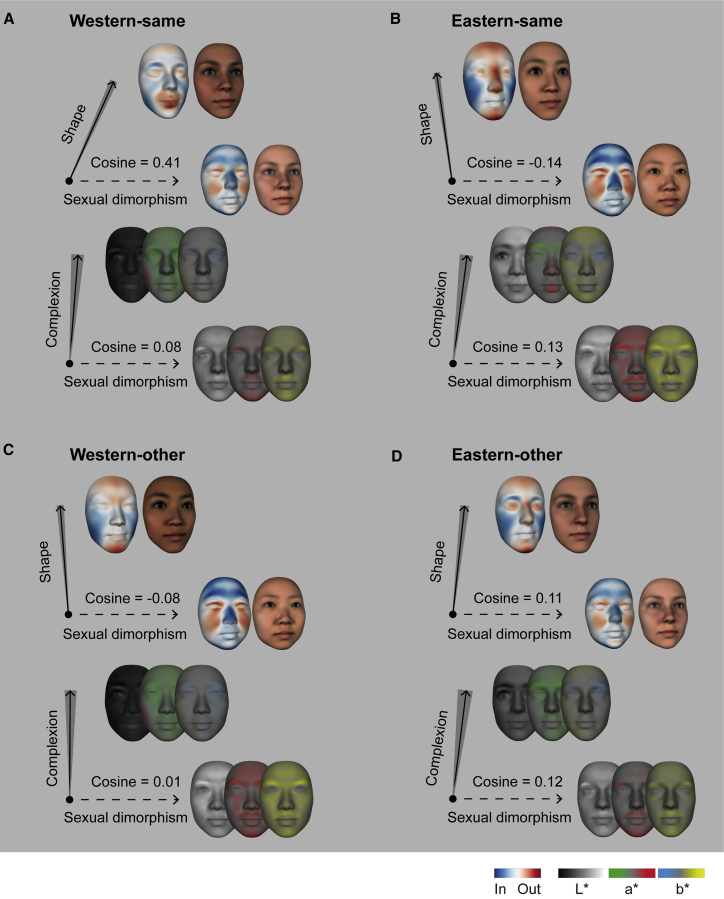
Facial attractiveness and sexual dimorphism are represented with different feature spaces (A) Western-same. Two directions of multivariate deviations (42 dimensional for shape and 116 dimensional for complexion) from the 25-year-old WE female average (black dot central to both axes) represent the Western-same attractiveness (solid arrow) and Western sexual dimorphism (dashed arrow), respectively. Adjacent faces (normalized color scale; to the maximum of each shape or L^∗^a^∗^b^∗^ display; sexual dimorphism amplified for display purposes) illustrate the multivariate contents of each axis, and their vector cosine quantifies their similarity relationship (vector cosine of 0 is orthogonality). Shaded regions flanking the solid arrows indicate the 95% confidence interval of the difference between attractiveness and sexual dimorphism. See also Figure S2 for the cosine similarity of each individual model. (B) Eastern-same, same format as (A). (C) Western-other, same format as (A). (D) Eastern-other, same format as (A).

Sexually dimorphic features make faces look more masculine or feminine and can indicate sexual maturity and reproductive potential.^24^, ^25^, ^26^ Contrary to existing theories, we show that sexual dimorphic and attractive face features differ. First, we computed for each face ethnicity the direction of shape and complexion deviations that transform the average male face into the average female face. Next, we compared these directions with those that make faces look more attractive (see Sexual dimorphism and attractiveness in the STAR Methods). In each condition of our design, directions of sexual dimorphism and attractiveness differed for both shape and complexion (vector cosine similarity < 0.4; p < 0.05; in some cases near orthogonal; see Figures 2A and 2B). Specifically, WE preferences for pouty mouths, darker skin, and redder cheeks and EA preferences for higher nose bridges, pointy chins, prominent foreheads, and high-contrast eyelids (denoted as solid arrows in Figures 2A and 2B) are each different from feminine deviations of high cheekbones, smaller foreheads and noses, and lighter skin (denoted as dashed arrows in Figure 2). Thus, shape and complexion deviations of facial attractiveness are not exaggerations of (i.e., colinear with) feminine sexual dimorphism (or masculine, the opposite direction). Instead, facial attractiveness evaluation (and perhaps evolution) is based on a different set of features (see Figure S2 for this analysis with individual models).

We repeated the analyses for Western and Eastern participants viewing other-ethnicity faces (henceforth, “Western-other” and “Eastern-other”) and replicated these key findings (Figures 1C, 1D, 2C, and 2D).

### Culture: Cultural commonalities, differences, and individual preferences

The analyses of feature selection suggest that facial attractiveness is represented by its own feature space that we now model. Within this space, we examine cultural commonalities and differences and diversity in individual preferences.

#### The representation space of facial attractiveness

**Figure 3:**
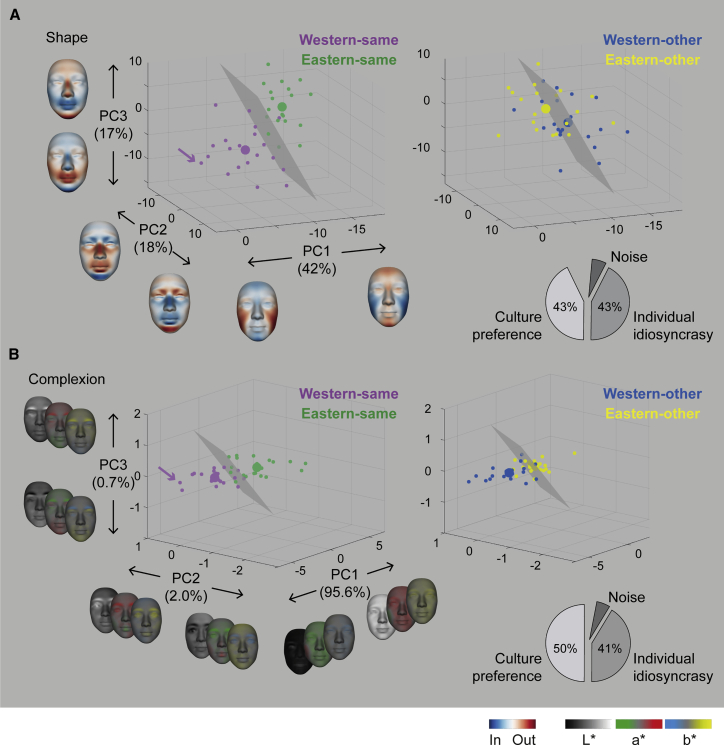
Cultural commonalities, differences, and individual preferences (A) Shape. Three axes of the space represent the first three components (i.e., principal components [PCs]) that capture the shape variance (74%) of individual participant models. Left: same-ethnicity: faces on each axis show the multivariate attractive feature of each component (normalized color scale to the maximum of each shape or L^∗^a^∗^b^∗^ display). Smaller dots represent the 40 individual models (Western-same, purple; Eastern-same, green); large dots represent their averages. Gray boundary surfaces (SVM classifier) separate Western-same from Eastern-same models, implying cultural specificity of attractive face shape features. Right: other-ethnicity: same as left is shown; blue and yellow dots denote individual Western-other and Eastern-other models, respectively. Pie chart shows the proportions of variance explained by the four group averages (i.e., a cultural preference) versus individuals’ idiosyncratic preference. See also Table S3. (B) L^∗^a^∗^b^∗^ complexion, same format as (A).

To derive a representational space of facial attractiveness, we applied a principal component analysis (PCA) to all 80 individual face models for shape (Figure 3A) and complexion (Figure 3B) separately (see Components of attractiveness in the STAR Methods). The first three components captured 74% of shape variance and 97% of complexion variance (see also Table S3). Figure 3 shows these components as separate axes. Each participant’s model is represented as a colored dot with coordinates indicating the strength of each component of a multivariate attractive feature (faces on each axis show their features). We then used the space to examine cultural commonalities, differences, participant idiosyncrasies, and their transference across face ethnicities.

#### Cultural commonalities and differences

To compare preferences across cultures, we used the individual participant models of the same-ethnicity conditions. Figures 3A (shape) and 3B (complexion), left panel, show that Western-same models (purple dots) form the cloud that is distinct from Eastern-same models (green dots). Though, in both cultures, most face models shared a smaller face and prominent forehead (i.e., negative values for shape PC1), separation of the purple and green dots with the gray boundary surface (SVM classifier) reveals marked cultural differences: Western-same models show poutier mouths and smaller noses (shape PC3) and darker skin and redder cheeks (complexion PC1); Eastern-same models show smaller mouths and higher nose bridges (shape PC3), narrower faces and pointier chins (shape PC1), and lighter, yellower skin (complexion PC1).

#### Individual differences

We also separated the variations of idiosyncratic preferences of individual participants from the variations explained by the group averages (see Decomposing preference variance in the STAR Methods). As shown by the pie charts on the right, cultural group models (large colored dots) and individual models (small colored dots) explained a similar amount of variance for shape and complexion. This demonstrates that individual preferences maintain variations of attractive features within each culture (see Germine et al.^9^ and Hönekopp^10^ for similar results with ratings of full-face stimuli).

#### Are cultural idiosyncrasies specific to own-ethnicity faces or are they pervasive, transferring across to other-ethnicity faces?

**Figure 4:**
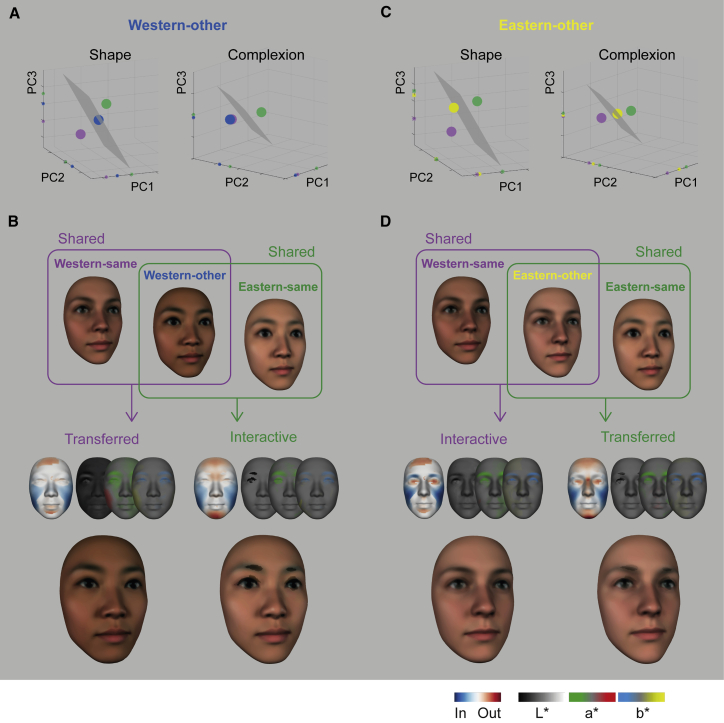
Transferred versus interactive attractiveness features for other-ethnicity faces (A) Western-other. The facial attractiveness representation space (shape, left panel; complexion, right panel) shows Western-other preferences (group average model, blue dot) relative to Western-same (group average model, purple dot) and Eastern-same (group average model, large green dot). On each axis, colored tick markers show the respective loading of each model. (B) Shared features between Western-same and Western-other in the purple set indicate transfer of attractive Western-same features to Western-other. Shared features between Western-other and Eastern-same in the green set indicate interactive preferences. Smaller faces show the transferred and interactive shape and complexion (color scale normalized to the maximum of each display). Larger faces visualize the transferred and interactive features. (C) Eastern-other, color-coded in yellow, same format as (A). (D) Shared features between Eastern-same and Eastern-other in the green set indicate transfer of attractive Eastern-same features to Eastern-other. Shared features between Western-same and Eastern-other in the purple set indicate interactive preferences. Same format as (B) is shown.

To address this question, we used group models in each culture (see Transferred and interactive preference in the STAR Methods). In Figure 4A (left panel), the intermediate location of Western-other shape preferences in the space (i.e., the blue dot located between the purple Western-same and the green Eastern-same preferences) indicates a transfer of Western-same preferences to other-ethnicity EA faces (e.g., a smaller shaped nose), together with the development of new interactive preferences when Westerners evaluate EA faces (e.g., preference for a pointier chin in EA faces). Figure 4B illustrates the respective contributions of transferred and interactive face feature preferences. Complexion in Figure 4A (right panel) reveals, with the overlap of the blue Western-other and the purple Western-same models, that WE complexion preferences fully transfer to EA faces (e.g., darker skin and redder cheeks). The same analysis applied to Eastern-other (yellow dot) in Figure 4C reveals transferred shape preferences for a higher nose bridge and pointy chin while reducing preferences for lighter skin complexion (see Figure 4D). In sum, all participants transferred their attractive face feature cultural preferences from same-ethnicity faces to other-ethnicity faces while also developing new interactive preferences.

Here, we revisited the long-standing question of what makes a face attractive? A prominent finding is that attractive face features are functional for mate choice, which in turn could put face shape and complexion under evolutionary selection pressures. According to one theory, attractive faces are closer to the population average because they reflect both developmental stability and genetic diversity.^27^^,^^28^ Another prominent theory is that attractive face features correlate with those of sexual dimorphism and show exaggerated secondary sexual characteristics that indicate high fertility and health in the context of mate selection.^24^, ^25^, ^26^ Theory-driven approaches have therefore played a major role in proposing and testing specific hypotheses about the nature of human preferences (see Thornhill and Gangestad,^29^ Rhodes,^30^ Little et al.,^31^ and Fink and Penton-Voak^32^ for review). However, they can also constrain the development of knowledge due to the cultural biases of researchers.^33^

Using a data-driven approach, we overcome these constraints to model the shape and complexion face features that drive attractiveness perceptions in two cultures and face ethnicities. Contrary to existing accounts, we found that attractive face features form a space that is distinct from both the average and from sexually dimorphic features (replicating Said and Todorov^6^ and Holzleitner et al.^7^). Critically, we show that attractive shape and complexion face features sit at the outskirts of the natural distribution of face variations, reflecting preferences for elaborate cues^34^—e.g., redder skin in Westerners, reflecting more oxygenated blood;^35^ super-stimulation (e.g., redder lips); and a yellower skin in EAs, reflecting a healthier, carotenoid-rich diet,^35^ plus a significant level of idiosyncrasies across individuals.

Such preferences could have evolved from biases in the face evaluation mechanisms of males who find specific, exaggerated female face features more attractive. Such biases could result in an evolutionary drift of peak preference toward exaggerated features in the directions that we modeled (see Ryan and Cummings^19^ and, for simulations, Arak and Enquist^18^ and Enquist and Arak^36^ in other contexts). We show that these exaggerated feature preferences also transfer to other-ethnicity faces coupled with additional interactive preferences. Other exaggerated features differed across cultures and individuals, which could reflect the influence of cultural and social factors in modern society, as is found with shape in visual perception generally^37^^,^^38^ (see Rhodes et al.^39^ for facial attractiveness). Thus, idiosyncratic preferences, at the cultural and individual level, could develop from variations in lifelong experiences, within a socio-cultural niche. Internationalization of social media could also contribute to statistical learning by strengthening cultural stereotypes. For example, our models revealed the current Western cultural stereotype preference for female pouty mouths and tanned skin and the EA cultural stereotype preferences for higher nose bridges and lighter skin.

To achieve such detailed characterization of face features, our modeling uniquely leveraged the power of the GMF, which belongs to the broad class of 3D morphable, active appearance models (AAMs) of facial synthesis.^40^ Specifically, AAMs represent the 3D surface and 2D complexion of faces as independent dimensions, which affords improved stimulus control compared to the 2D-image-based face spaces typically used to study facial attractiveness. Though other AAMs have been used to model facial attractiveness,^6^^,^^41^ the GMF affords further, tighter control of the categorical factors of age, sex, ethnicity, and individual identity, whose face shape and complexion variances are explained in the model. Feature characterization is a necessary hallmark of psychophysical, data-driven studies of social trait perception (see Jack and Schyns^42^ for discussions). It is necessary to understand how the participant’s psychology—here, their perception of facial attractiveness—changes systematically with changes of physical stimulus properties—here, the mathematically modeled face features. Feature characterization is also important (and a unique application of our approach) to transfer these understandable models to socially interactive digital agents to enhance their artificial intelligence in generating culturally suitable signs of facial attractiveness and better engage their human users (see Chen et al.^43^^,^^44^).

Mathematical characterization of attractive face features revealed cultural diversity, which, though derived from small sub-populations, logically negates theories of universal standards. Evidence of cultural diversity raises the broader question of how the multiple factors of sex,^13^, ^14^, ^15^, ^16^ age,^45^, ^46^, ^47^ hormonal cycle,^48^, ^49^, ^50^, ^51^, ^52^, ^53^ social trait,^54^^,^^55^ short- versus long-term relationship prospects,^56^, ^57^, ^58^ culture, and others could influence perception of facial attractiveness. Our data-driven framework can address such questions and characterize and compare the features potentially associated with each factor. Such extensive, suitably controlled studies could document a new, dynamic theory of feature spaces that evolve over the lifespan in line with the evolving preferences of the cultural individual.^59^ In turn, such models would enable digital agents to adjust their social signaling capabilities across user lifespans.

To better model attractive face features, the GMF could control hairstyles and associated features of hair color, length, and texture. Enhanced experimental designs could also add dynamic facial expressions and coloration (e.g., blushing) to reveal possible interactions between external features (e.g., hair),^60^^,^^61^ facial expression,^62^^,^^63^ and facial attractiveness judgments. Finally, our stimuli were synthesized and the data acquired in the laboratory. The acid test of the validity of our models is whether they would generalize with impact to real-world faces and tasks, which we have demonstrated previously with facial expression models applied to a socially interactive digital agent.^43^^,^^44^ In the context of face identity, our models elicited accurate identifications, even when the faces were changes in age, sex, or viewpoint.^17^ Thus, future work should test the transfer of our mathematical models of attractiveness to such real-world situations.

To conclude, using a data-driven method, we modeled the shape and complexion face features that drive perceptions of attractiveness between and within cultures and their interaction with face ethnicity. Our results directly inform and impact fundamental theories of facial attractiveness in human psychology and evolutionary biology by providing the specific representational contents of individual preferences across cultures and revealing their commonalities and diversities. Our approach opens new avenues to understand the nature of facial attractiveness and other subjective social perceptions in the culturally diverse social world.

## STAR★Methods

### Key resources table

**Table:** 

REAGENT or RESOURCE	SOURCE	IDENTIFIER
**Deposited data**		

Raw and analyzed data	This paper	https://dx.doi.org/10.17632/cvh2d2bz6r.2

**Software and algorithms**		

MATLAB R2015b & R2020b	Mathworks	RRID: SCR_001622
Psychtoolbox-3	http://psychtoolbox.org/	RRID: SCR_002881
Custom Code for analyses	This paper	https://dx.doi.org/10.17632/cvh2d2bz6r.2

### Resource availability

#### Lead contact

Further information and requests for resources should be directed to and will be fulfilled by the Lead Contact Philippe G. Schyns (Philippe.Schyns@glasgow.ac.uk)

#### Materials availability

This study did not generate new unique reagents.

#### Data and code availability

Original and analyzed data reported in this study have been deposited to Mendeley Data: https://doi.org/10.17632/cvh2d2bz6r.2. The code for analyses has been deposited to Mendeley Data: https://doi.org/10.17632/cvh2d2bz6r.2. The code for GMF modeling, experiment and visualization is available by request to the Lead Contact.

### Experimental model and subject details

#### Participants

We recruited a total of 80 male participants (40 Western European, WE, and 40 East Asian, EA, mean and median age = 23, SD = 2.93), who self-reported heterosexual preferences. Our sample size aligned with this literature.^6^^,^^7^^,^^64^ A questionnaire assessed that all WEs had minimal experience of non-Western cultures and all EAs had resided in the UK for < 6 months, with limited prior exposure to non-Eastern cultures (see Questionnaire in the STAR Methods). All participants had normal or corrected to normal vision, with no self-reported history or symptoms of synesthesia, and/or any psychological, psychiatric or neurological condition that can affect face processing (e.g., depression, Autism Spectrum Disorder or prosopagnosia). Participants gave written informed consent prior to testing and received ≤6 per hour for their participation. The University of Glasgow College of Science and Engineering Ethics Committee provided ethical approval.

#### Questionnaire

##### Western European Participants

Each potential Western European participant answered the following questionnaire. We selected only those 1) who answered ‘no’ to all questions, or 2) who answered ‘no’ to question 2 &3 and ‘yes’ to question 1 but had traveled to non-Western^∗^ country for only a short vacation (i.e., < 2 weeks).

1 How long you have spent in a non-Western^∗^ country IN TOTAL since you were 10 years old?
2 Have you ever been in close contact with any non-Western^∗^ person(s) who has been your friend or acquaintance for quite some time?
3 Have you ever been involved with any non-Western^∗^ culture societies/groups?
∗ by Western groups/countries, we are referring to Europe (Eastern and Western), USA, Canada, United Kingdom, Australia and New Zealand.

##### East Asian Participants

Each potential East Asian participant answered the following questionnaire. We selected those who entered the UK < 6 months (question 1), spent < 6 months in a non-Eastern culture (question 2), and answered ‘no’ to question 3 and 4.

1 At what date did you first enter the UK?
2 How long you have spent in a non-Eastern^∗^ country IN TOTAL since you were 10 years old?
3 Have you ever been in close contact with any non-Eastern^∗^ person(s) who has been your friend and acquaintance for quite some time?
4 Have you ever been involved with any non-Eastern^∗^ culture societies/groups?
∗ by Eastern groups/countries, we are referring to China, Japan, Korea, Thailand and Taiwan.

### Method details

#### 3D Face Stimuli

We used our Generative Model of 3D Faces (GMF^17^) to synthesize 3,900 random 25-year-old female faces equally split between WE and EA ethnicities. We set female stimuli to 25-year of age, associated with high fertility and likely to convey features of facial attractiveness to males of a similar age,^29^^,^^65^ to control age-related female face variations. Our GMF controls 3D face-identity variance using a database of 467 3D faces (see GMF 3D Face Database in the STAR Methods). The GMF decomposes a 3D face (parametrized with 4,735 3D vertex coordinates for shape and 800 × 600 RGB pixels for complexion, see Figure S1A) into two components: a categorical average defined by factors of face age (i.e., set to 25 years old), ethnicity (WE versus EA) and sex (set to female), plus a residual component that identifies each generated face. Two linear transformations underlie the generative model: (1) the extraction of a categorical average 3D face (represented by 4,735×3 shape coordinates and 800×600×3 complexion pixels) that shares the features of 25-year-old WE or EA females and (2) a Principal Components Analysis (PCA) of the multivariate residuals (as 4,735×3 shape vertices and 800×600×3 complexion pixels) that represent identity-specific features as a 467×1 shape vector coefficients (one per principal component) and a 467×5 matrix of complexion coefficients across 5 spatial frequency (SF) bands (again, one coefficient per principal component). To generate each face, we generated random identity residuals (separately for shape and complexion), by multiplying the generative PCs with random coefficients and added the categorical average of each ethnicity (i.e., WE or EA, see Figure S1B). At this stage, it is critical to understand that we added same set of 1,950 random identity residuals to the WE and EA female categorical averages, so that WE and EA stimuli share the same age, sex and random identity variations and only differed in their average ethnic information.

#### GMF 3D Face Database

The face database comprised 172 Western Caucasian females, 124 Western Caucasian males, 90 East Asian females, 74 East Asian males, 3 Black African females, 4 Black African males, age between 16 and 88, SD = 13, scanned in-house with a Di4D face capture system, at a high resolution in shape (4,735 3D vertex coordinates) and texture (800×600 RGB pixels, see Figure S1A). All 3D models were in full color with hair removed, posing with a neutral facial expression.

#### Procedure

Each trial started with a central fixation cross displayed for 1 s, followed by a face presented on a black screen subtending an average of 9.5°×6.4° of visual angle, until response. We instructed participants to quickly rate the attractiveness of the face, based on their first impression, with a mouse click and using a 9-point rating scale displayed under the face (1, *not attractive at all*; 9, *very attractive*). Following response and a 500 ms blank interval a new trial would begin. The experiment comprised 1,950 trials in a 2×2 between-participants design, so that each cultural participant (EA or WE) would rate faces only from one ethnicity (i.e., either WE or EA faces). Across trials, the 1,950 3D stimuli appeared on the screen presented in one of three evenly distributed viewpoints (−30, 0 and +30° of rotation in depth). The experiment comprised a total of 39 randomly allocated blocks of 50 trials that each participant performed over 2 to 3 days. Participants sat in a dimly lit room and used a chin rest to maintain a fixed viewing distance. We used the Psychtoolbox^66^ for MATLAB R2018a to control the experiment.

### Quantification and statistical analysis

#### Linear Regression Model

We performed linear regression analyses independently for each participant. An experimental trial paired the trial-specific stimulus parameters (a 467-dimensional vector of random shape coefficients; a 467 × 5 dimensional matrix of random complexion coefficients) with the corresponding participant’s attractiveness rating response (a value between 1 and 9). Across trials, we linearly regressed the stimulus parameters with the participant’s ratings, separately for each shape and complexion dimension (RobustFit, MATLAB 2018a) as in Equation 1 below.

(Equation 1) $${\text{PC}}_{\text{j}}\text{coefficient}\;=\text{β}1\;+\text{β}2\;\text{Ratings}$$

Equation 1 delivered a linear model with β1 and β2 coefficients for each dimension of 3D shape and 2D complexion that generated the face stimuli. These β1 and β2 coefficients therefore model and explain how variations of face shape and complexion linearly relate to variations of facial attractiveness perception in each participant. We called these β1 s and β2 s the participant’s model of facial attractiveness. This model is multivariate, comprising 467 shape dimensions and 467 × 5 complexion dimensions, separately for the β1 and β2 coefficients that multiply the principal components of shape and complexion.

We repeated these regression analyses at the finer granularity of individual shape vertices and complexion pixels, to address with univariate analyses effects that the multivariate analyses might hide. For each shape vertex (N = 4,735), we linearly and independently regressed its X, Y and Z 3D face coordinates on the corresponding attractiveness ratings; likewise, for each of complexion pixel (N = 480,000 down-sampled to 30,000 pixels), we linearly and independently regressed its L^∗^, a^∗^, b^∗^ channels on the corresponding attractiveness ratings (p < 0.05, two-tailed, for the β2 s, corrected for multiple comparisons with the false discovery rate method,^67^ across all 4735^∗^3 vertex coordinates and 200^∗^150^∗^3 L^∗^a^∗^b^∗^ pixel values). Figure S2 report these individual models, showing high correspondence between the multivariate and the univariate linear regressions.

We checked the linear assumption prior to linear modeling, with a three-step procedure that we first illustrate with shape features:

Step 1: In each experimental condition, we computed the average face across the face stimuli corresponding to each one of 5 attractiveness rating bins.
Step 2: To quantify how the average face of each attractiveness bin (from Step 1) deviated from the categorical average of our GMF (i.e., the average female WE or EA face in 25 years old), we computed the vertex-wise distances between the average face in each rating bin and the GMF categorical average face. This delivered a 4,735 × 5 distance matrix (4735 face vertices in each one of 5 rating bins
Step 3: To summarize changes of the 4,735 vertices between bin 1 to bin 5, we applied a k-means clustering to the 4,735^∗^5 distance matrices obtained from Step 2. This revealed mainly 4 patterns of vertex-wise changes (number of clusters was determined by the elbow method, *cf.* small panel in each line plot). The lines plot in Figures S2A-3–S2D-3 show these patterns with the centroid of each k-means cluster. We can see that the shape changes are near-linear from bin 1 (unattractive faces) to bin 5 (attractive faces). We applied the same analyses to face complexion, independently for L^∗^a^∗^b^∗^ pixels (see Figures S2A-3–S2D-3), resulting also in the suitability of a linear assumption to model the overall relationship between changes of complexion and changes of perceived attractiveness.

Thus, in each of our four experimental conditions, linear changes of shape vertices and L^∗^a^∗^b^∗^ pixels related to linear changes in face attractiveness, justifying our modeling of the relationships with linear regressions.

Furthermore, we modeled, independently for each participant, the relationship between each shape/complexion PC coefficient and participants’ ratings with Mutual Information (MI), which can capture any relationship (i.e., linear *and* nonlinear) between any pair of variables.^68^ Resulting models were highly similar between linear regression and MI model reconstructions, as we now detail. Figures S2A-3–S2D-3 show the MI models of each group average, where we signed the MI values with the positive or negative slope of the beta coefficients in the linear regression models for comparison purposes. Linear regression models (see Figures 1 and S2) and MI models (see Figure S2) were highly similar. To quantify the similarity, for shape vertices (i.e., 4,735-length vertex vectors), we computed the Spearman Rank correlation between linear regression and MI models and found significant correlation for all 4 conditions (*r* = 0.99, p < 0.05, one-side). For complexion L^∗^a^∗^b^∗^ (i.e., down-sampled to 30,000-length pixel vector for each color channel), Spearman Rank correlation between linear regression and MI models are also significant (p < 0.05 for all conditions, one-side), with *r* > 0.95 for all conditions, except *r* = 0.87 for a^∗^ in Eastern-same condition. We obtained null distributions for the above statistics by randomly shuffling, in each of 200 iterations, the MI across shape/complexion PCs, while keeping the same sign as the slopes of the beta coefficients to compute the Spearman Rank correlation. We calculated the 95 percentiles of the null distributions as threshold. These similarities between linear regression models and MI warranted linear modeling.

#### Reconstructing Attractive and Unattractive Faces

In the linear regression model of Equation 1, β2 coefficients control perceived facial attractiveness at the level of individual participants. Remember that our experiment comprised 4 conditions (Western-same and other; Eastern-same and other). For each condition, we computed a group-level model by averaging the β1 and β2 coefficients (of the multivariate linear regressions) of each individual participant. We then rendered positively (for attractive) and negatively (for unattractive) amplified shape and complexion β2 s of each group average (see Panel 7 and 9 in Figure 1).

#### Face Average and Feature Distribution

The GMF represents each rated stimulus as a categorical average plus an identity residual component, separately for shape and complexion (see 3D Face Stimuli in the STAR Methods). Here, in four steps we addressed the question of the location of attractive features in the distribution of shape and complexion variations, separately for each of our 4 conditions.

Step 1: We selected for each participant the stimuli rated as highly attractive and pooled these across all 20 participants of the considered experimental condition to form the attractive stimulus set.
Step 2: We extracted, for each shape and complexion PC, the identity coefficient that approximates the modal value of all identity coefficients in the attractive set from Step 1. We used the modal coefficient vector (i.e., 467×1 for shape and 467×5 for complexion identity residuals) plus corresponding categorical average to synthesize the face that describe the attractive features (i.e., 4,735 shape vertices and 800x600 complexion pixels’ L^∗^a^∗^b^∗^) of these attractive stimuli.
Step 3: We computed the mean and standard deviation of each face vertex and complexion across the 1,950 experimental stimuli.
Step 4: We z-scored attractive features per vertex and pixel L^∗^a^∗^b^∗^ that computed in Step 2, using their mean and standard deviations computed in Step 3.

#### Sexual Dimorphism and Attractiveness

In the group model, the 467 × 1 β2 s of shape and 467 × 5 β2 s of complexion specify multivariate directions of shape and complexion change (in the GMF PC space) that characterize an attractive female face away from the categorical average. To examine the relationship between this direction of change (for shape and complexion) and that of femininity (i.e., sexual dimorphism) we proceeded as follows. First, we computed the direction of sexual dimorphism as the per-vertex (for shape) and per pixel (for complexion) difference between average female and average male faces, independently for WE and EA faces. Second, we projected this male-female difference into the GMF PC space to reparametrize sexual dimorphism as a 467 × 1 shape vector and a 457 × 5 complexion matrix. Next, now that both attractiveness and sexual dimorphism were two directions in the same space, we computed their similarity with vector cosine. Specifically, we reduced the dimensionality of the vector cosine computation (using the elbow method, see Elbow Method in the STAR Methods in below) to keep only the eigenvectors with significant eigenvalues—i.e., for shape, 42 dimensions; for complexion 116 dimensions split as follows: 10 dimensions for SF1; 18 for SF2; 30 for SF3; 41 for SF4; 17 for SF5.

We further tested the inference that changes of facial attractiveness are represented along a direction significantly different from changes of sexual dimorphism. Specifically,

Step 1: For each participant, we computed 1,000 bootstrapped models (c.f. Linear Regression Model in the STAR Methods), sampling with replacement 1,950 trials, reducing the dimensions of these shape (and complexion) models to 42 (and 116), as above.
Step 2: We computed the vector cosine similarity of each bootstrapped model to the original model (see Linear Regression Model in the STAR Methods). From the resulting null distribution of 1000 vector cosines, we computed the 95% confidence interval (2.5 to 97.5 percentile in the distribution) of no significant difference. An original model of facial attractiveness model was significantly different from sexual dimorphism if its cosine similarity was smaller than the lower bound of the 95% confidence interval.

We repeated Step 1 and Step 2 independently for each individual model, separately for shape and complexion and each was significantly different as defined. For illustration purposes, in each experimental condition we averaged 20 individual models using the bootstrap iterations of each to derive a distribution of 1,000 group models of shape (and 1,000 group models of complexion). We used these group distributions to estimate the 95% group-level confidence interval of attractiveness versus sexual dimorphism dissimilarity plotted in Figure 2.

#### Components of Attractiveness

To characterize the representation space, we applied PCA to the 80 individual models of facial attractiveness, separately for shape and complexion, vectorizing the models at vertex- and pixel-level granularities—i.e., 14,205-dimensional shape vectors, from 4,735 vertices × 3 coordinates and 140,000-dimensional complexion vectors, from 800 × 600 pixels × 3 dimensions of L^∗^a^∗^b^∗^. Table S3 reported the variance explained by each significant shape PC (N = 8, explained 94.23% of total variance) and each significant complexion PC (N = 3, explained 98.32% of total variance), using the elbow method for significance (see Elbow Method in the STAR Methods in below). We used this space of significant PCSs for subsequent analyses.

#### Decomposing Preference Variance

In the space, we projected each model onto the 8 shape and 3 complexion PC dimensions, using the resulting scores for variance decomposition analyses into that explained: 1) by mean experimental condition (i.e., the cultural preference), and 2) by each individual model (i.e., the individual preference).

To do this, we used bootstrap resampling to quantify the sampling variance of the individual models obtained with our experimental procedure. We calculated 20 bootstrap models for each participant (c.f. Linear Regression Model in the STAR Methods) randomly sampling 1,950 trials with replacement. Then, we repeated the PCA analysis as in Components of Attractiveness in the STAR Methods, this time using 1,600 models (i.e., 80 participants × 20 bootstrapped models = 1600). Next, we decompose the variance of each shape and complexion component as follows:

Step 1: We calculated the total variance $SS_{total}$ of the attractiveness effect, using the sum of squared distance from 0 that quantifies no effect.
Step 2: We removed the corresponding group mean of the component and calculated the sum of squared residuals $SS_{resi1}$ not explained by the 4 group means. The proportion of variance (i.e., the $R^{2}$) explained by group means is given by:

(Equation 2) $$R_{group}^{2}=1\;-SS_{resi1}/SS_{total}$$

Step 3: We then removed, the participant-level residual mean, and calculated the SS of further left residuals $SS_{resi2}$ not explained by 80 participants’ means. The proportion of variance explained by individual model thus equals to (4):

(Equation 3) $$R_{participant}^{2}==SS_{resi1}/SS_{total}\;\times \left(1-SS_{resi2}/SS_{resi1}\right)$$

Across 8 shape components (and 3 complexion components, separately), we calculated the weighted sum of $R_{group}^{2}$ and $R_{participant}^{2}$ scaled by the variance explained by each PC and obtained overall in the attractiveness PC space the proportion of culture versus individual preferences (see pie chart in Figure 3). The remaining unexplained variance is related to the sampling variance of our individual models, revealed by the bootstrap resampling.

To determine the threshold of $R_{group}^{2}$ for each attractiveness dimension, in each of 200 iterations, we shuffled 1600 models across 4 conditions while keeping in each participant their bootstrapped models, and repeated Step 2 above. The 200 $R^{2}$ outcomes capture the null distribution of no group difference. We computed 99.375 percentile of the null as the shape threshold $R_{group}^{2}$, as we corrected the multiple comparison for 8 shape PCs (one-tailed, p < 0.05, Bonferroni corrected—i.e., 99.375 = 100 – 5/8). Likewise, we computed the 98.34 percentile of 200 $R^{2}$ outcomes for complexion $R_{group}^{2}$ (one-tailed, p < 0.05, Bonferroni corrected—i.e., 98.34 = 100 – 5/3). Across dimensions (8 for shape and 3 for complexion), we computed the weighted sum of the thresholds to obtain the overall threshold to determine significance and found significant $R_{group}^{2}$ for both shape and complexion.

To calculate the threshold of $R_{subj}^{2}$, we kept the 4 conditions in their right place, but shuffled all 400 models in each condition. We repeated Step3 in each of 200 iterations and calculated the threshold in the same way as we did for $R_{group}^{2}$. We found significant $R_{subj}^{2}$ for both shape and complexion (one-tailed, p < 0.05, Bonferroni corrected).

#### Transferred and Interactive Preference

To separate the respective contributions of transferred and interactive features in Western-other and Eastern-other models, we proceeded as follows. First, we computed the group models of each experimental condition by averaging the β2 coefficients across the 20 participants per condition in vertex and pixel L^∗^a^∗^b^∗^ spaces. For each vertex of (and pixel L^∗^a^∗^b^∗^) of the Western-other (and Eastern-other) group model we computed whether it had the same sign as the Western-same model and, independently, as the Eastern-same model. A transferred feature of Western-other (and Eastern-other) would share β2 signs with Western-same (versus Eastern-same). An interactive feature of Western-other (versus Eastern-other) would share β2 signs with Eastern-same (versus Western-same).

#### Model Validation

We validated each individual model, using their shape and complexion projection in the representation space of attractiveness, to predict the per-trial attractiveness judgements of individual participants. We did so to test that our representation space better predicts participant’s ratings than the face average and the sexual dimorphism hypothesis.

For each participant, we randomly segmented the full trials (N = 1,950) into 13 blocks and performed a 13-folds cross validation. In each of the 13 iterations, we proceeded in 4 steps, separately for shape and complexion:

Step 1: We built a linear regression model using trials in 12 adjacent blocks (i.e., training set, N = 1,800 trials), as in Linear Regression Model in the STAR Methods.
Step 2: We used the model from Step1 and another 79 models of left out participants (from above Linear Regression Model in the STAR Methods) to derive the space of attractiveness (same as Components of Attractiveness in the STAR Methods).
Step 3: For each stimulus, we extracted its identity residual by removing the GMF categorical average and reparametrized the residuals into the attractiveness space (as an 8-dimensional shape vector and a 3-dimensional complexion vector) and used these as the predictor X. We defined Y as the attractiveness rating to be predicted based on X (separately for shape and complexion).
Step 4: We trained a Generalized Linear Model (GLM) to predict the attractiveness ratings Y from the predictors X—i.e., Y’ = exp(X*b*). We used the log link function with a Gamma error distribution to account for the non-negative and skewed ratings (only few trials were rated as highly attractive). We fitted the GLM model using the method of iterative reweighted least-squares implemented in fitglm in MATLAB 2019a. We tested the model prediction performance using the trials in the leave-out block (N = 150) and checked the ranking consistency (Kendall τ rank correlation) between each participant’s actual ratings and the GLM predicted ratings.

For comparison, we also trained 2 alternative GLM models:

Alternative model 1 (averageness): the predictor X for each stimulus was the global distance of its shape (or complexion) to the GMF face average of its experimental condition, where we computed shape global distance as the Euclidean distance between the stimulus position to the center of the space (i.e., the GMF average face, a distance between two 467-dimensional shape coefficients). We repeated this distance computation for complexion in the vectorized 2,335-dimension GMF complexion space (2,335 = 467×5).
Alternative model 2 (sexual dimorphism): the predictor X for each stimulus was the scalar projection of its GMF shape PCs (or complexion PCs) onto the shape (or complexion) sexual dimorphism vector (see sexual dimorphism calculation as above). The vector scalar projection was computed using a reduced set of GMF PCs, i.e., for shape, 42 dimensions; for complexion 116 dimensions (10 dimensions for SF1; 18 for SF2; 30 for SF3; 41 for SF4; 17 for SF5), defined by the elbow method as we did above.

We also trained and tested the alternative models in a 13-folds cross-validation. Figure S3 shows the prediction performance quantified by Kendall’s τ rank correlation of all models, in which we showed our attractiveness models predict subjective ratings significantly better than average (pair-wised t test, p < 0.001 for both shape and complexion, Bonferroni correction) and sexual dimorphism models (pair-wised t test, p < 0.001 for both shape and complexion, Bonferroni correction).

#### Elbow Method

##### Sexual Dimorphism and Attractiveness

We ranked the eigenvalues of GMF PCs and plotted the eigenvalue curve (see Figure S4A, black curve). We determined the threshold point (see red point in Figure S4A) on the curve that has the furthest distance (see d’ in Figure S4A) from the straight line connecting the first and last point of the eigenvalue curve (see dash line in Figure S4A). Figure S4B shows the feminine features represented by the significant components against the representations of the full components, demonstrating the reliability of this method.

##### Components of Attractiveness

We ranked the eigenvalues of attractiveness PCs and determined the threshold point as we introduced above (c.f. Figure S4A), separately for shape and complexion. Figure S4C shows the attractive features represented by the significant components against the representations of the full components, demonstrating the reliability of this method.

## Supplemental information

Document S1. Figures S1–S4 and Tables S1–S3

Document S2. Article plus supplemental information
