# Supplementary material for: Modeling individual preferences reveals that face beauty is not universally perceived across cultures
Source: Curr Biol. 2021 May 24;31(10):2243–2252.e6. doi: 10.1016/j.cub.2021.03.013 (PMC8162177; doi:10.1016/j.cub.2021.03.013)
Supplement: Document S1. Figures S1–S4 and Tables S1–S3 [file mmc1.pdf]

Current Biology, Volume *31*

Supplemental Information

Modeling individual preferences reveals that face beauty is not universally perceived across cultures

Jiayu Zhan, Meng Liu, Oliver G.B. Garrod, Christoph Daube, Robin A.A. Ince, Rachael E. Jack, and Philippe G. Schyns

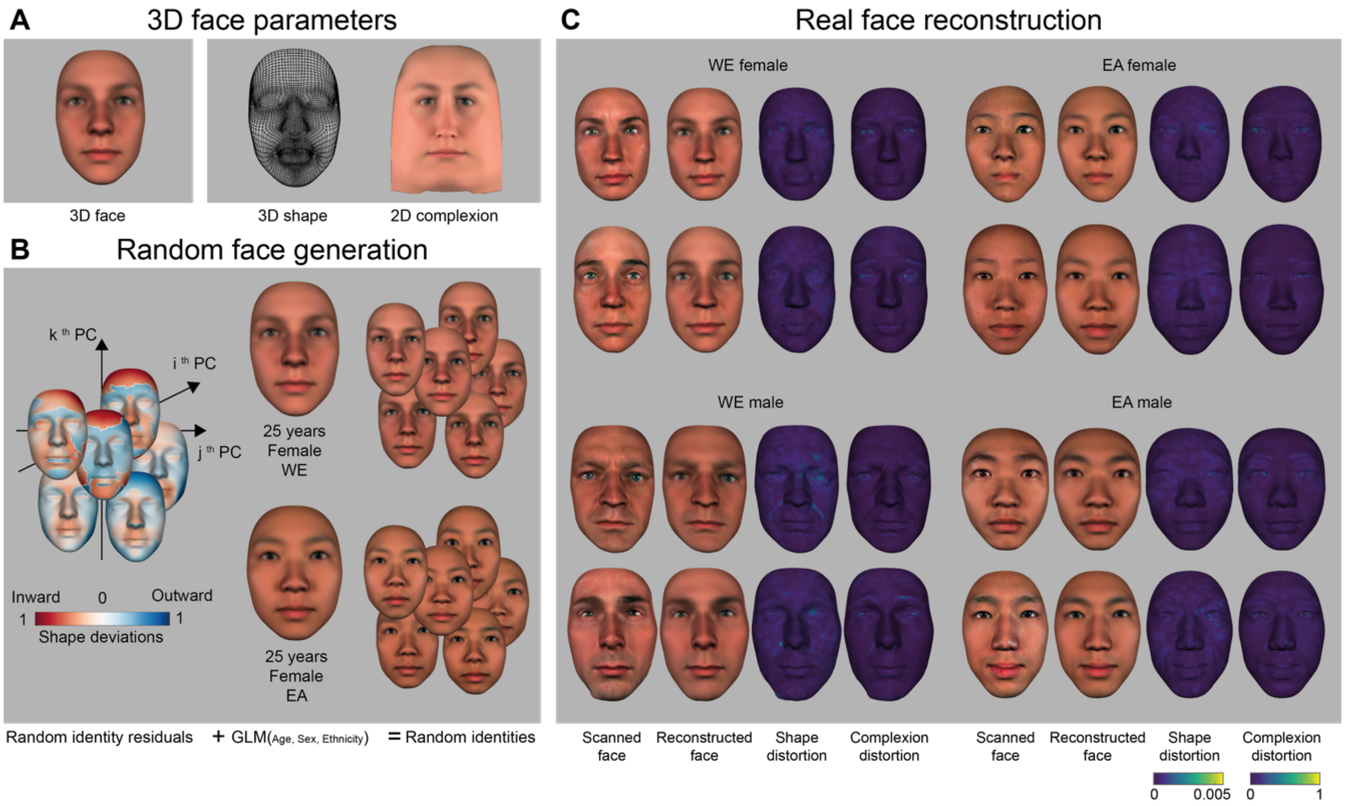

Figure S1. Generative Model of 3D Face Identity (GMF). Related to STAR METHODS, METHOD DETAILS, 3D Face Stimuli.

(A) 3D face parameters. We parametrized the shape of a 3D face with the 3D coordinates of 4,735 vertices and its complexion with 800×600×3 RGB 2D pixels.

(B) Random face generation. In the GMF, we synthesized each face stimulus as the sum of a categorical average capturing the factors of face sex (female), ethnicity (WE or EA), age (25 years old) and their interactions plus a random component of 3D shape identity (and 2D complexion component, not illustrated here). Across the experiment, all face stimuli shared the same categorical average and had a unique and randomly defined identity. The color scale indicates the magnitude of 3D shape deviations from the categorical average (normalized by the maximum deviation).

(C) Real face reconstruction. To demonstrate accuracy of reconstruction of novel faces (and therefore expressiveness of natural variations in the GMF), we scanned 8 new faces and fitted each within the GMF. The low distortion magnitudes indicate, vertex per vertex (and pixel per pixel), the high quality of the reconstructed GMF fit to the ground truth scanned faces which indicates that it can represent (i.e., generate) face variations beyond the sample that it has been trained on. For shape vertices, distortions are calculated in Euclidean space (scanned face – reconstructed face)/reconstructed face. For complexion pixels, distortions are calculated as (scanned face – reconstructed face)/reconstructed face, separately for L*a*b* channels. The panel reports the averaged distortions across 3 color channels. We obtained informed consent from these 8 persons for the use of their faces in publications.

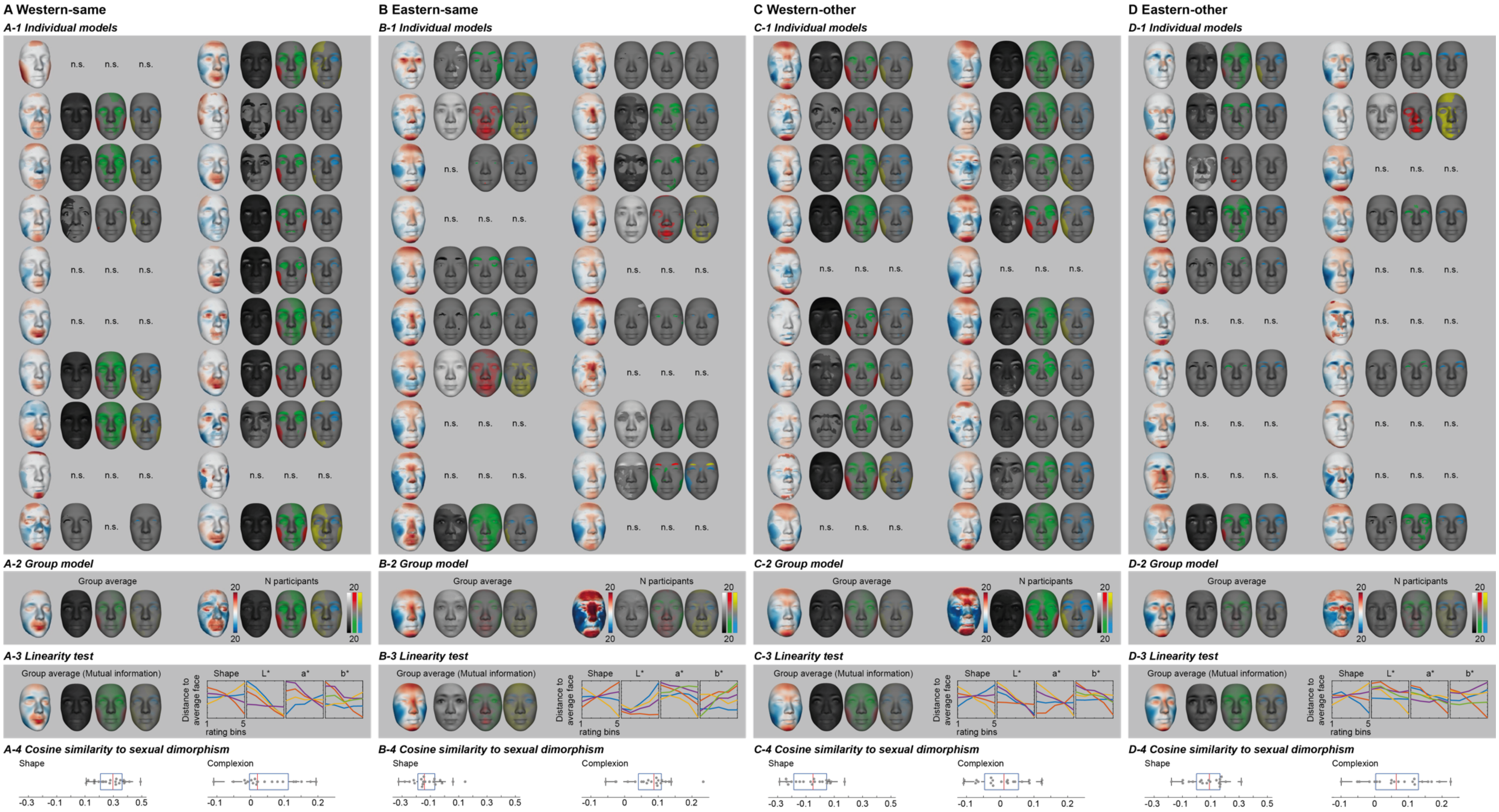

Figure S2. Individual Models. Related to Figure 1, Figure 2 and STAR METHODS, QUANTIFICATION AND STATISTICAL ANALYSIS, Linear Regression Model.

(A) Western-same models. *(A-1) Individual models.* For each participant, we colored significant shape vertices and L*a*b* pixels according to their effect size (i.e., slope β in the linear model, normalized by each display maximum). *(A-2) Group model.* Left panel shows averaged effect sizes across participants (color scale normalized to the maximum of each display). Right panel shows number of participants, per vertex and pixel L*a*b*, with significant β slope. *(A-3) Linearity test*. Left panel faces show the averaged MI models (MI signed with β slopes) across participants (color scale normalized to the maximum of shape or L*a*b*). Colored lines in the right panel show the main clusters of the vertex-wise or pixel-wise distances (y-axis) between the average face in each rating bin (x-axis) and the GMF categorical average, separately for shape and complexion. *(A-4) Cosine Similarity to Sexual Dimorphism.* Cosine similarity between sexual dimorphism and individual facial attractiveness of shape (left panel) and complexion (right panel). Boxplots show the cosine similarity of each individual model (overlaid as solid dots). n.s. = not significant.

(B) Eastern-same models, same caption as (A).
(C) Western-other models, same caption as (A).
(D) Eastern-other models, same caption as (A).

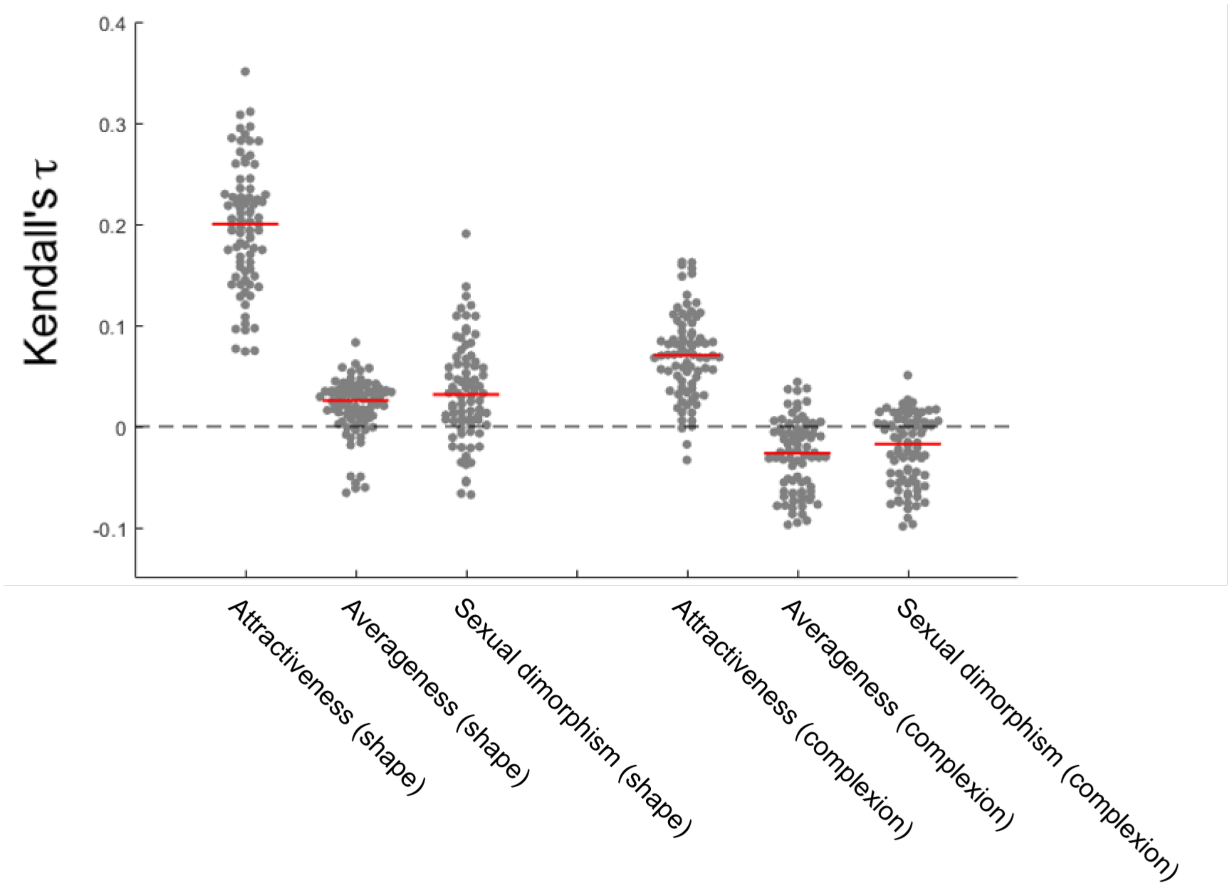

Figure S3. Model Validation. Related to STAR METHODS, QUANTIFICATION AND STATISTICAL ANALYSIS, Model Validation.
Prediction performance (y-axis) of each tested GLM model (x-axis), measured by the Kendall's τ correlation between each participant's actual ratings vs. GLM predicted ratings. Red lines denote models' median prediction performance across 80 participants.

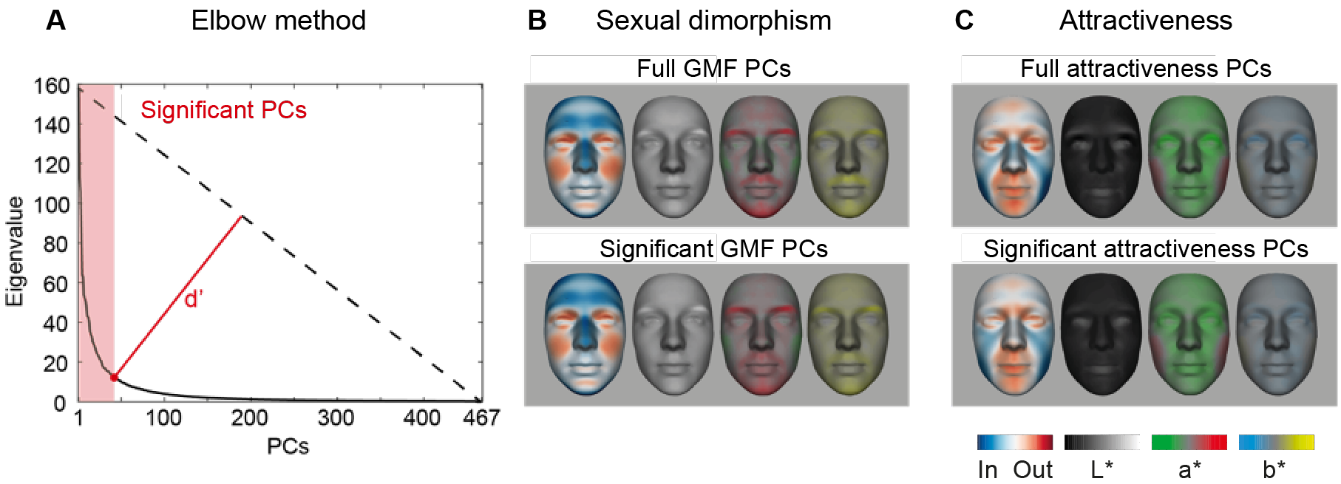

Figure S4. Elbow Method. Related to STAR METHODS, QUANTIFICZTION AND STATISTICAL ANALYSIS, Elbow Method.

(A) Elbow method. The elbow point (red point) on the Eigenvalue curve (black curve) has the largest distance (d') to the dash line connecting the first and last PCs. PCs explained significant variance are those before the elbow point.

(B) Sexual dimorphism. Comparison of Full GMF PCs' representation (first row) and the representation of the significant PCs determined by the elbow method (second row), separately for shape and L*a*b* complexion. Colored scales on the face indicate the magnitude of shape (normalized to the maximum) and L*a*b* deviations (normalized to the maximum across three channels).

(C) Attractiveness, same as (B). GMF = Generative Model of 3D Face.

Features	Dimensions	Positive		Negative	
		Western same	Papers	Western same	Papers
Forehead prominence	Shape	Displayed	"S1", "S2"	--	--
Eye size in general	Shape	Displayed	"S1", "S5"	--	--
Eye width	Shape	Displayed	"S6", "S7", "S8", "S9", "S10", "S11"	--	--
Eye height	Shape	Displayed	"S6", "S7", "S12"	--	--
Outer eye corner width	Shape	Displayed	"S2", "S3"	--	--
Mouth size in general	Shape	Displayed	"S2", "S9", "S11", "S13"	--	--
Mouth height	Shape	Displayed	"S9", "S10"	--	--
Mouth protruding	Shape	Displayed	"S14", "S15", "S16"	--	--
Upper lip thickness	Shape	Displayed	"S1", "S4", "S9", "S13", "S14"	--	--
Lower lip thickness	Shape	Displayed	"S1", "S2", "S4", "S7", "S11", "S13", "S14"	--	--
Chin prominence	Shape	Displayed	"S15"	--	--
Cheekbone prominence	Shape	Displayed	"S1", "S6", "S7", "S8", "S9"	--	--
Eyebrow darkness	Complexion	Displayed	"S1"	--	--
Contrast in eye region	Complexion	Displayed	"S1", "S17", "S18", "S19", "S20"	--	--
Redness of skin	Complexion	Displayed	"S23", "S24"	--	--
Nose size in general	Shape	--	--	displayed	"S1", "S6", "S7", "S9"
Nose width	Shape	--	--	displayed	"S9"
Nose length	Shape	--	--	displayed	"S3", "S4", "S9", "S10", "S12"
Chin area in general	Shape	--	--	displayed	"S1", "S9"
Chin width	Shape	--	--	displayed	"S7", "S9"
Chin length	Shape	--	--	displayed	"S4", "S6", "S7"
Face width at mouth	Shape	--	"S3"	displayed	"S1", "S6", "S7"
Luminance of skin	Complexion	--	--	displayed	"S1", "S22"
Forehead height	*Shape*	*NA*	*"S3", "S4"*	*NA*	--
Distance between eyes	*Shape*	*NA*	*"S1", "S6", "S7"*	*NA*	--
Eyebrow height	*Complexion*	*NA*	*"S6", "S7"*	*NA*	*"S11"*
Eyebrow thickness	*Complexion*	*NA*	--	*NA*	*"S1", "S7", "S9"*
Contrast in mouth region	*Complexion*	*n.s.*	*"S17", "S19"*	*n.s.*	--
Redness of lip	*Complexion*	*n.s.*	*"S22"*	*n.s.*	--

Table S1. External Validation of Western-same Model. Related to Figure 1A.

The western-same model is highly consistent with the attractive features reported in the literature. Positive (vs. Negative) denotes the positive (vs. negative) feature changes in relation to increased attractiveness. Features listed in Italics are reported in papers, but not applicable (NA)/measurable or not significant in our models. n.s. = not significant. Numbers correspond to the index of the Supplemental references.

Features	Dimensions	Positive		Negative	
		Eastern same	Papers	Eastern same	Papers
Forehead prominence	Shape	displayed	“S25”	--	--
Eye size in general	Shape	displayed	“S11”, “S26”	--	--
Eye width	Shape	displayed	“S7”, “S11”, “S25”	--	--
Eye height	Shape	displayed	“S7”, “S11”, “S25”, “S27”	--	--
Nose length	Shape	displayed	“S25”	--	--
Nose bridge height	Shape	displayed	“S26”, “S28”, “S29”	--	--
Nose tip height	Shape	displayed	“S15”, “S25”	--	--
Chin prominence	Shape	displayed	“S15”, “S25”	--	--
Chin length	Shape	displayed	“S28”, “S29”, “S34”	--	“S7”, “S25”
Contrast in eye region	Complexion	displayed	“S26”	--	--
Contrast in mouth region	Complexion	displayed	“S26”	--	--
Nostril width	Shape	--	--	displayed	“S25”, “S26”
Mouth height	Shape	--	--	displayed	“S29”
Mouth protruding	Shape	--	--	displayed	“S30”, “S31”, “S32”, “S33”
Upper lip thickness	Shape	--	--	displayed	“S15”, “S19”
Lower lip thickness	Shape	--	--	displayed	“S15”, “S19”
Chin width	Shape	--	--	displayed	“S7”, “S26”, “S29”, “S34”
Cheekbone prominence	Shape	--	--	displayed	“S25”, “S28”, “S29”, “S34”
Face width at cheekbone	Shape	--	--	displayed	“S25”, “S28”, “S34”
Face width at mouth	Shape	--	--	displayed	“S25”, “S28”, “S29”, “S34”
Luminance of skin	complexion	--	--	displayed	“S26”, “S35”, “S36”
Forehead height	*Shape*	*NA*	*“S25”*	--	--
Nose size in general	*Shape*	*NA*		*NA*	*“S7”*
Eyebrow height	*Complexion*	*NA*	--	*NA*	*“S11”*
Eyebrow thickness	*Complexion*	*NA*	--	*NA*	*“S26”*

Table S2. External Validation of Eastern-same Model. Related to Figure 1B.
The eastern-same model is highly consistent with the attractive features reported in the literature. Same caption as Table S1.

Shape		Complexion	
PC	Explained variance	PC	Explained variance
1	41.46%	1	96.60%
2	17.53%	2	2.01%
3	16.74%	3	0.73%
4	6.77%	--	--
5	4.90%	--	--
6	3.07%	--	--
7	2.21%	--	--
8	1.37%	--	--

Table S3. Variance Explained by Each Attractive Component. Related to Figure 3 and STAR METHODS, QUANTIFICATION AND STATISTICAL ANALYSIS, Components of Attractiveness.

Supplemental References

S1. C. Braun, M. Gründl, C. Marberger, C. Scherber, Beautycheck - causes and consequences of attractiveness. (2001).

S2. C. Sforza, *et al.*, Soft-Tissue Facial Characteristics of Attractive Italian Women as Compared to Normal Women. *Angle Orthod* **79**, 17–23 (2009).

S3. V. F. Ferrario, C. Sforza, C. E. Poggio, G. Tartaglia, Facial morphometry of television actresses compared with normal women. *Journal of Oral and Maxillofacial Surgery* **53**, 1008–1014 (1995).

S4. V. S. Johnston, M. Franklin, Is beauty in the eye of the beholder? *Ethology and Sociobiology* **14**, 183–199 (1993).

S5. S. Geldart, D. Maurer, K. Carney, Effects of Eye Size on Adults' Aesthetic Ratings of Faces and 5-Month-Olds' Looking Times. *Perception* **28**, 361–374 (1999).

S6. M. R. Cunningham, Measuring the Physical in Physical Attractiveness: Quasi-Experiments on the Sociobiology of Female Facial Beauty. *Journal of Personality and Social Psychology* **50**, 925–935 (1986).

S7. M. R. Cunningham, A. R. Roberts, A. P. Barbee, P. B. Druen, C.-H. Wu, "Their ideas of beauty are, on the whole, the same as ours": Consistency and variability in the cross-cultural perception of female physical attractiveness. *Journal of Personality and Social Psychology* **68**, 261–279 (1995).

S8. K. Grammer, R. Thornhill, Human (Homo sapiens) facial attractiveness and sexual selection: The role of symmetry and averageness. *Journal of Comparative Psychology* **108**, 233–242 (1994).

S9. J.-Y. Baudouin, G. Tiberghien, Symmetry, averageness, and feature size in the facial attractiveness of women. *Acta Psychologica* **117**, 313–332 (2004).

S10. D. Jones, *et al.*, Sexual Selection, Physical Attractiveness, and Facial Neoteny: Cross-cultural Evidence and Implications [and Comments and Reply]. *Current Anthropology* **36**, 723–748 (1995).

S11. S. C. Rhee, K.-S. Woo, B. Kwon, Biometric Study of Eyelid Shape and Dimensions of Different Races with References to Beauty. *Aesth Plast Surg* **36**, 1236–1245 (2012).

S12. L. Z. McArthur, K. Apatow, Impressions of Baby-Faced Adults. *Social Cognition* **2**, 315–342 (1984).

S13. M. Bisson, A. Grobbelaar, The Esthetic Properties of Lips: A Comparison of Models and Nonmodels. *Angle Orthod* **74**, 162–166 (2004).

S14. T. A. Auger, P. K. Turley, The female soft tissue profile as presented in fashion magazines during the 1900s: a photographic analysis. *Int J Adult Orthodon Orthognath Surg* **14**, 7–18 (1999).

S15. H. S. Oh, *et al.*, Correlations between cephalometric and photographic measurements of facial attractiveness in Chinese and US patients after orthodontic treatment. *American Journal of Orthodontics and Dentofacial Orthopedics* **136**, 762.e1-762.e14 (2009).

S16. S. Matoula, H. Pancherz, Skeletofacial Morphology of Attractive and Nonattractive Faces. *Angle Orthod* **76**, 204–210 (2006).

S17. R. Russell, Sex, Beauty, and the Relative Luminance of Facial Features. *Perception* **32**, 1093–1107 (2003).

S18. R. Mulhern, G. Fieldman, T. Hussey, J.-L. Lévêque, P. Pineau, Do cosmetics enhance female Caucasian facial attractiveness? *International Journal of Cosmetic Science* **25**, 199–205 (2003).

S19. N. L. Etcoff, S. Stock, L. E. Haley, S. A. Vickery, D. M. House, Cosmetics as a Feature of the Extended Human Phenotype: Modulation of the Perception of Biologically Important Facial Signals. *PLOS ONE* **6**, e25656 (2011).

S20. D. Peshek, N. Semmaknejad, D. Hoffman, P. Foley, Preliminary Evidence that the Limbal Ring Influences Facial Attractiveness. *Evol Psychol* **9**, 147470491100900200 (2011).

S21. I. D. Stephen, A. M. McKeegan, Lip Colour Affects Perceived Sex Typicality and Attractiveness of Human Faces. *Perception* **39**, 1104–1110 (2010).

S22. A. G. Miller, W. A. Ashton, J. W. McHoskey, J. Gimbel, What Price Attractiveness? Stereotype and Risk Factors in Suntanning Behavior. *Journal of Applied Social Psychology* **20**, 1272–1300 (1990).

S23. B. Fink, K. Grammer, R. Thornhill, Human (Homo sapiens) facial attractiveness in relation to skin texture and color. *Journal of Comparative Psychology* **115**, 92–99 (2001).

S24. A. D. Pazda, C. A. Thorstenson, A. J. Elliot, D. I. Perrett, Women's Facial Redness Increases Their Perceived Attractiveness: Mediation Through Perceived Healthiness. *Perception* **45**, 739–754 (2016).

S25. K. S. Jang, *et al.*, A three-dimensional photogrammetric analysis of the facial esthetics of the Miss Korea pageant contestants. *Korean J Orthod* **47**, 87 (2017).

S26. K. Nakamura, K. Watanabe, Data-driven mathematical model of East-Asian facial attractiveness: the relative contributions of shape and reflectance to attractiveness judgements. *R. Soc. open sci.* **6**, 182189 (2019).

S27. L. Z. McArthur, D. S. Berry, Cross-Cultural Agreement in Perceptions of Babyfaced Adults. *Journal of Cross-Cultural Psychology* **18**, 165–192 (1987).

S28. Q. Zhao, *et al.*, Morphological Quantitative Criteria and Aesthetic Evaluation of Eight Female Han Face Types. *Aesth Plast Surg* **37**, 445–453 (2013).

S29. S. Samizadeh, W. Wu, Ideals of Facial Beauty Amongst the Chinese Population: Results from a Large National Survey. *Aesth Plast Surg* **42**, 1540–1550 (2018).

S30. J. Soh, M. T. Chew, H. B. Wong, An Asian community's perspective on facial profile attractiveness. *Community Dentistry and Oral Epidemiology* **35**, 18–24 (2007).

S31. J. Soh, M. T. Chew, H. B. Wong, Professional assessment of facial profile attractiveness. *American Journal of Orthodontics and Dentofacial Orthopedics* **128**, 201–205 (2005).

S32. H. Ioi, T. Shimomura, S. Nakata, A. Nakasima, A. L. Counts, Comparison of anteroposterior lip positions of the most-favored facial profiles of Korean and Japanese people. *American Journal of Orthodontics and Dentofacial Orthopedics* **134**, 490–495 (2008).

S33. S. Kuroda, *et al.*, Influence of anteroposterior mandibular positions on facial attractiveness in Japanese adults. *American Journal of Orthodontics and Dentofacial Orthopedics* **135**, 73–78 (2009).

S34. S. C. Rhee, S. H. Lee, Attractive Composite Faces of Different Races. *Aesth Plast Surg* **34**, 800–801 (2010).

S35. B. J. Dixson, A. F. Dixson, B. Li, M. J. Anderson, Studies of human physique and sexual attractiveness: Sexual preferences of men and women in China. *American Journal of Human Biology* **19**, 88–95 (2007).

S36. C. Han, *et al.*, Cultural differences in preferences for facial coloration. *Evolution and Human Behavior* **39**, 154–159 (2018).
